# Target gene approaches: Gene expression in *Daphnia magna *exposed to predator-borne kairomones or to microcystin-producing and microcystin-free *Microcystis aeruginosa*

**DOI:** 10.1186/1471-2164-10-527

**Published:** 2009-11-16

**Authors:** Anke Schwarzenberger, Cornelius Courts, Eric von Elert

**Affiliations:** 1 University of Cologne, Zoological Institute, Aquatic Chemical Ecology, Weyertal 119, 50923 Cologne, Germany; 2 University Hospital Cologne, Department of Neuropathology, Kerpener Str. 62, 50937 Cologne, Germany; 3 University Hospital Bonn, Institute for Forensic Medicine, Stiftsplatz 12, 53111 Bonn, Germany

## Abstract

**Background:**

Two major biological stressors of freshwater zooplankton of the genus *Daphnia *are predation and fluctuations in food quality. Here we use kairomones released from a planktivorous fish (*Leucaspius delineatus*) and from an invertebrate predator (larvae of *Chaoborus flavicans*) to simulate predation pressure; a microcystin-producing culture of the cyanobacterium *Microcystis aeruginosa *and a microcystin-deficient mutant are used to investigate effects of low food quality. Real-time quantitative polymerase chain reaction (QPCR) allows quantification of the impact of biotic stressors on differential gene activity. The draft genome sequence for *Daphnia pulex *facilitates the use of candidate genes by precisely identifying orthologs to functionally characterized genes in other model species. This information is obtained by constructing phylogenetic trees of candidate genes with the knowledge that the *Daphnia *genome is composed of many expanded gene families.

**Results:**

We evaluated seven candidate reference genes for QPCR in *Daphnia magna *after exposure to kairomones. As a robust approach, a combination normalisation factor (NF) was calculated based on the geometric mean of three of these seven reference genes: *glyceraldehyde-3-phosphate dehydrogenase, TATA-box binding protein *and *succinate dehydrogenase*. Using this NF, expression of the target genes *actin *and *alpha-tubulin *were revealed to be unchanged in the presence of the tested kairomones. The presence of fish kairomone up-regulated one gene (*cyclophilin*) involved in the folding of proteins, whereas *Chaoborus *kairomone down-regulated the same gene.

We evaluated the same set of candidate reference genes for QPCR in *Daphnia magna *after exposure to a microcystin-producing and a microcystin-free strain of the cyanobacterium *Microcystis aeruginosa*. The NF was calculated based on the reference genes *18S ribosomal RNA*, *alpha-tubulin *and *TATA-box binding protein*. We found *glyceraldehyde-3-phosphate dehydrogenase *and *ubiquitin conjugating enzyme *to be up-regulated in the presence of microcystins in the food of *D. magna*. These findings demonstrate that certain enzymes of glycolysis and protein catabolism are significantly upgregulated when daphnids ingest microcystins. Each differentially regulated gene is a member of an expanded gene family in the *D. pulex *genome. The *cyclophilin*, *GapDH *and *UBC *genes show moderately large sequence divergence from their closest paralogs. Yet *actin *and *alpha-tubulin *genes targeteted by our study have nearly identical paralogs at the amino acid level.

**Conclusion:**

Gene expression analysis using a normalisation factor based on three reference genes showed that transcription levels of *actin *and *alpha-tubulin *were not substantially changed by predator-borne chemical cues from fishes or invertebrates, although changes in expression on the protein level were shown elsewhere. These changes in protein level could be caused by others than the investigated paralogs, showing the importance of the construction of phylogenetic trees for candidate gene approaches. However, fish kairomones caused an up-regulation, and *Chaoborus *kairomone caused a down-regulation of *cyclophylin*, which proved to be a potential target gene for further analysis of kairomone effects on the life history of daphnids. Changes in food quality required a different set of reference genes compared to the kairomone experiment. The presence of dietary microcystins led to an up-regulation of two genes involved in the basic metabolism of *D. magna*, i.e. *glyceraldehyde-3-phosphate dehydrogenase *and *ubiquitin conjugating enzyme*, which suggests that microcystins in cyanobacteria have more general effects on the metabolism of *D. magna *than previously thought. Phylogenetic trees resolving relationships among paralogs that share the same gene name are shown to be important for determining the identity of the candidate genes under investigation.

## Background

Notwithstanding other so called 'model organisms', of which whole genome sequences have been obtained, the ecology of the model organism *Daphnia *sp. is outstandingly well known. Therefore it is a challenge to investigate gene/environment interactions for major ecological interactions of this cladoceran. In most freshwater lakes and ponds, *Daphnia *sp. is the major consumer of algae and cyanobacteria and is also the most important prey for predatory invertebrates and planktivorous fishes. In line with its intermediate position in the food chain, *Daphnia *sp. populations can be controlled by predation (top-down) or resources (bottom-up). However, the impact of top-down and bottom-up factors on *Daphnia *sp. population dynamics shows a pronounced seasonality [1]. Predation pressure is low in spring, but peaks with the appearance of young-of-the-year fishes and fourth-instar larvae of *Chaoborus *water midges in early summer and remains moderate until fall [2,3]. Bottom-up factors become a major constraint on *Daphnia *sp. population growth, particularly in eutrophic lakes in the summer when mass developments of toxic cyanobacteria lead to a suppression of *Daphnia *sp. biomass [4,5]. The low predictability of intensity and seasonality of both predation pressure and dominance of toxic cyanobacteria should lead to the evolution of plastic instead of fixed adaptations [6]. Indeed, adaptive phenotypic plasticity in *Daphnia magna *to both toxic cyanobacteria and predator-borne chemical cues has been reported [7-14]. *Daphnia magna *has been shown to reduce size at first reproduction (SFR) in response to kairomones from fish whereas chemical cues from larvae of *Chaoborus flavicans *led to increased SFR; both responses have been proven to be adaptive as fishes and *Chaoborus *differ in size-selectivity of their prey [10-14]. A different kind of phenotypic plasticity constitutes the enhanced tolerance of *D. magna *against cyanobacterial toxins upon exposure to a toxin producing cyanobacterium. The recent release of the *Daphnia pulex *genome sequence (wFleaBase: http://wFleaBase.org, JGI Genome Portal: http://www.Jgi.doe.gov/Daphnia/) creates the opportunity to precisely identify candidate genes that differ in their expression in response to predator-borne chemical cues (i.e. kairomones) and to a toxic cyanobacterium as a first step to decipher the underlying molecular mechanisms of adaptive phenotypic plasticity in *D. magna*.

Toxin production is a characteristic feature of several strains of the bloom-forming freshwater cyanobacterium *Microcystis aeruginosa*. A large variety of cyclic heptapeptides, termed microcystins (which can become a health hazard to humans and livestock [15]), have been identified in *M. aeruginosa*. Wild type *M. aeruginosa *PCC7806 produces relatively large amounts of two microcystin variants (LR and RR); experiments with a microcystin-free mutant of this strain [16] have led to the conclusion that microcystins contribute to the daphnid poisoning by *M. aeruginosa *[17,18].

The microcystins of *M. aeruginosa *PCC7806 are known to inhibit protein phosphatases 1 and 2A in warm-blooded animals and in *Daphnia *sp [19]., which suggests that the poisoning effect of microcystin-LR in *Daphnia *sp. is due to the inhibition of these two protein phosphatases. However, it remains entirely unclear which major physiological pathways in *Daphnia *sp. are affected by the binding of microcystin to protein phosphatases 1 and 2A.

For single clones of *D. magna *it has been shown that they develop tolerance against a microcystin producing cyanobacterium [20,21]. Although the physiological mechanisms have not been elucidated, this increased tolerance can be transferred to the offspring via maternal effects [20]. For a better understanding of the process of physiological adaptation of daphnids to toxic cyanobacteria, it is important to examine the genes that are differentially regulated in the presence of microcystins. To achieve this goal we quantified the expression of a set of genes involved in the basic metabolism in *D. magna *when cultured on a *M. aeruginosa *PCC7806 mutant in which the production of microcystins had been knocked out, or on the microcystin-synthesizing wild type strain. As a reference cyanobacterium, we used a strain of *Synchecoccus elongatus *which is non-toxic to daphnids [22] and easily ingested; a strain of the green alga *Scenedesmus obliquus *which is widely used as standard food for daphnids was used as reference for high quality food [23].

Much of the recent evidence for inducible defences in freshwater ecology which has contributed to the general understanding of predator-prey interactions has been derived from experimental studies with daphnids. Despite considerable progress in the understanding of inducible defences, the underlying plasticity has rarely been studied at a molecular level. Only recently has the response of *D. magna *to kairomones from fish and invertebrates been investigated on the protein level, and it has been shown that a clone of *D. magna *that was isolated from a habitat where it coexists with fishes and invertebrate predators responds with a decrease in the proteins actin and alpha-tubulin [24]. We hypothesized that this decrease might be due to a change in transcription of the *actin *and *alpha-tubulin *genes and that these genes might function as targets for predator-borne chemical cues. We therefore exposed the same clone of *D. magna *as Pijanowska & Kloc (2004) [24] to similar kairomones and quantified the transcription levels of putative target genes.

## Results

### Kairomone experiment: Effects on the life-history of *Daphnia magna*

**Figure 1:**
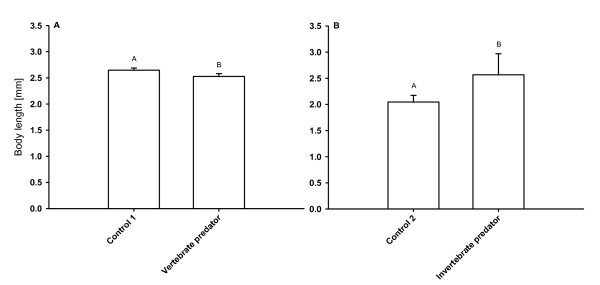
**Size at first reproduction of *Daphnia magna *in the kairomone experiment**. A: *D. magna *was grown either in water exposed to *Leucaspius delineatus *(sunbleak, 'Vertebrate predator') or in water without kairomone (control 1), or B: either in water exposed to larvae of *Chaoborus flavicans *('Invertebrate predator') or in the respective kairomone-free water (control 2). Depicted is the size at first reproduction (n = 3, ± SD). Letters indicate a significant difference (p < 0.001) between treatments.

In two different life-history experiments the size at first reproduction (SFR) of *D. magna *grown in water exposed either to sunbleaks (*Leucaspius delineatus*) or larvae of *Chaoborus flavicans *was determined. The SFR of *D. magna *grown in fish incubation water was significantly lower than SFR of the control group (p < 0.001, Tukey HSD after one-way ANOVA: F_8;0.00195 _= 33023.42; Fig. 1A), whereas SFR of *D. magna *raised in *Chaoborus *incubation water was significantly higher than SFR of *D. magna *grown in kairomone-free water (p < 0.001, Tukey HSD after one-way ANOVA: F_13;0.08364 _= 949.3778; Fig 1B).

### Kairomone experiment: Identification of reference genes and normalisation factors

**Figure 2:**
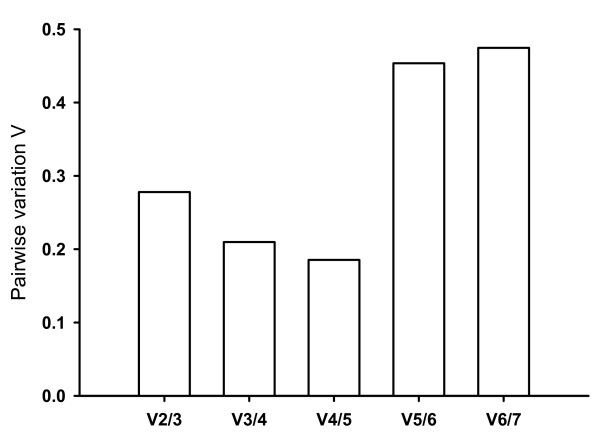
**Pair wise variation of sequential normalisation factors (Vn/n+1) in the kairomone experiment estimated by geNorm**. V2/3 is based on the geometric mean of the normalisation factors of *GapDH*, *TBP *and *SucDH*; V3/4 is V2/3 and *cyclophilin*; V4/5 is V3/4 and *UBC*; V5/6 is V4/5 and *28S*; V6/7 is V5/6 and *18S*.

In the kairomone experiment, the relative expression of the six candidate reference genes in the different treatments was analysed by geNorm (see Methods) and ranked according to increasing variability (*GapDH = SucDH < TBP < cyclophilin < UBC < 28S < 18S*). GeNorm calculated five normalisation factors. Pair wise comparison of sequential normalisation factors showed a low level of variability between the three most stable reference genes (V2/3; Fig. 2). In accordance with the recommendation of Vandesompele et al. (2002) [25] to use a minimum number of three reference genes, the normalisation factor generated from the three least variable genes (*GapDH, SucDH, TBP*) was used for normalisation in further analyses. The normalized values of the three reference genes showed little variation across treatments, resulting in low values of SD (Tab. 1, 2).

**Table 1:** Relative gene expression of the reference genes in *D. magna *after normalisation in the fish-kairomone experiment.

	Relative expression^a^		
Gene	Calibrator	Vertebrate predator	SD^b^
*SucDH*	1.0000 ± 0.0051	1.123 ± 0.0179	0.0867

*GapDH*	1.0000 ± 0.0078	1.145 ± 0.0073	0.1027

*TBP*	1.0000 ± 0.0063	0.7778 ± 0.0164	0.1571

*D. magna *was raised either in a predator-free environment ('Calibrator') or in incubation water of *Leucaspius delineatus *(sunbleak, 'Vertebrate predator').

^a^Values are mean of n = 3 replicates ± SD. Expression levels are displayed relative to the mean control level

^b^SD is the variation of one reference gene across treatments.

**Table 2:** Relative gene expression of the reference genes in *D. magna *after normalisation in the *Chaoborus*-kairomone experiment.

	Relative expression^a^		
Gene	Calibrator	Invertebrate predator	SD^b^
*SucDH*	1.0000 ± 0.0024	1.4451 ± 0.0059	0.3147
*GapDH*	1.0000 ± 0.0035	0.8881 ± 0.0113	0.0792
*TBP*	1.0000 ± 0.0018	0.7792 ± 0.0058	0.1561

*D. magna *was raised either in a predator-free environment ('Calibrator'), or in incubation water of larvae of *Chaoborus flavicans *('Invertebrate predator').Values are mean of n = 3 replicates ± SD.

^a^Expression levels are displayed relative to the mean control level

^b^SD is the variation of one reference gene across treatments

### Kairomone experiment: Relative normalized expression of the target genes *actin *and *alpha-tubulin*

**Figure 3:**
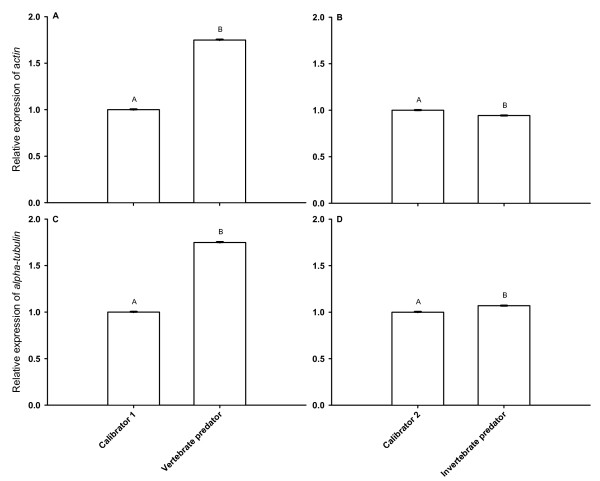
**Relative expression of the target genes *actin *and *alpha-tubulin *in *Daphnia magna *in the kairomone experiment**. *D. magna *was raised either in a vertebrate predator-free environment ('Calibrator1'), in incubation water of *Leucaspius delineatus *(sunbleak, 'Vertebrate predator'; A and C), or either in the respective calibrator ('Calibrator2') or in incubation water of larvae of *Chaoborus flavicans *('Invertebrate predator'; B and D). Depicted is the mean relative expression (n = 3, ± SD) of *actin *or *alpha-tubulin*. Letters indicate a significant difference (p < 0.001) between calibrator and kairomone treatments.

The mean relative expression of *actin *in the fish-kairomone treatment was significantly higher than in the respective control (Tukey's HSD post-hoc, p < 0.001 after one-way ANOVA: F_9;0.00001 _= 2037412, p < 0.001; Fig. 3A), whereas the mean relative expression of *actin *was significantly lower in the *Chaoborus*-kairomone treatment than in its respective control (Tukey's HSD post-hoc, p < 0.001 after one-way ANOVA: F_9;0.00001 _= 2037412, p < 0.001; Fig. 3B). Chemical cues from fishes led to a 1.75-fold increase in the relative expression of *actin *(Fig. 3A), and chemical cues from *Chaoborus *larvae decreased *actin *expression 0.94-fold (Fig. 3B). The standard deviation of the relative expression of *actin *across fish-kairomone treatments was 0.53 and was thus around four orders of magnitude higher than SD in the reference genes (Tab. 1). The standard deviation of the relative expression of *actin *across *Chaoborus*-kairomone treatments was 0.04 and had thus almost the same SD across treatments as the reference genes (Tab. 2).

A different response was found in the mean relative expression of *alpha-tubulin*. There was a significant 1.7-fold increase between fish-kairomone treatment and control (Tukey's HSD post-hoc, p < 0.001 after one-way ANOVA: F_9;0.00026 _= 64420.31, p < 0.001; Fig. 3C), and also a significant 1.07-fold increase of the mean relative expression of *alpha*-*tubulin *between control and *Chaoborus*-kairomone treatment (Tukey's HSD post-hoc, p < 0.001 after one-way ANOVA: F_9;0.00026 _= 64420.31, p < 0.001; Fig. 3D). The standard deviation of the relative expression of *alpha-tubulin *across fish-kairomone treatments was 0.50 and was thus around four orders of magnitude higher than SD in the reference genes (Tab. 1). The standard deviation of the relative expression of *alpha-tubulin *across *Chaoborus*-kairomone treatments was 0.05 and had thus almost the same SD across treatments as the reference genes (Tab. 2).

### Kairomone experiment: Relative normalized expression of non-reference genes

The non-reference genes in the fish-treatments (*28S*, *UBC*, *18S, cyclophilin*) showed values of SD across treatments (Tab. 3) that were at least four orders of magnitude higher than those of the normalised values of the three reference genes (*SucDH, GapDH*, *TBP*, SD across treatments < 0.158; Tab. 1). The same non-reference genes in the *Chaoborus*-treatment showed values of SD across treatments (Tab. 4) that were at least 1.3 orders of magnitude higher than those of the normalised values of the three reference genes (SD across treatments < 0.32; Tab. 2) with one exception (*28S*, SD across treatments = 0.15; Tab. 4). These findings suggested a treatment-dependent expression. All non-reference genes showed significantly different expression between the fish-treatment (Tab. 3), the *Chaoborus *treatment and their respective controls (*28S*: p < 0.001, Tukey HSD after one-way ANOVA: F_9;0.000212 _= 712.2765; *UBC*: p < 0.001, Tukey HSD after one-way ANOVA: F_9;0.000012 _= 30550.68; *18S*: p < 0.001, Tukey HSD after one-way ANOVA: F_9;0.000014 _= 32074.06; *cyclophilin*: p < 0.001, Tukey HSD after one-way ANOVA: F_9;0.000003 _= 8773865; Tab. 4), which indicated kairomone-dependent expression. The most striking effect was the up-regulation of *cyclophilin *(2.9-fold) by fish kairomone (Tab. 3) and its down-regulation (0.4-fold) by *Chaoborus *kairomone (Tab. 4). The other non-reference genes were all up-regulated in the kairomone treatments relative to their respective control, with a stronger effect of fish kairomone (1.86 - 2.25-fold; Tab. 3) than of *Chaoborus *kairomone (1.21 - 1.82-fold; Tab. 4).

**Table 3:** Relative gene expression of the non-reference genes in *D. magna *after normalisation in the fish-kairomone experiment.

	Relative expression^a^		
Gene	Calibrator	Vertebrate predator	SD^b^
*18S*	1.0000 ± 0.0123	2.2449 ± 0.0278	0.8803
*28S*	1.0000 ± 0.0519	1.8555 ± 0.0873	0.6049
*cyclophilin*	1.0000 ± 0.0012	2.9216 ± 0.0020	1.3588
*UBC*	1.0000 ± 0.0046	2.0671 ± 0.0251	0.7546

*D. magna *was raised in either a predator-free environment ('Calibrator') or in incubation water of *Leucaspius delineatus *(sunbleak, 'Vertebrate predator').

^a^Values are mean of n = 3 replicates ± SD. Expression levels are displayed relative to the mean control level

^b^SD is the variation of one reference gene across treatments

**Table 4:** Relative gene expression of the non-reference genes in *D. magna *after normalisation in the Chaoborus kairomone experiment.

	Relative expression^a^		
Gene	Calibrator	Invertebrate predator	SD^b^
*18S*	1.0000 ± 0.0033	1.8183 ± 0.0069	0.5786
*28S*	1.0000 ± 0.0065	1.2141 ± 0.0013	0.1514
*cyclophilin*	1.0000 ± 0.0012	0.3877 ± 0.0022	0.4329
*UBC*	1.0000 ± 0.0042	1.7394 ± 0.0042	0.5229

*D. magna *was raised in either a predator-free environment ('Calibrator'), or in incubation water of larvae of *Chaoborus flavicans *('Invertebrate predator').

^a^Values are mean of n = 3 replicates ± SD. Expression levels are displayed relative to the mean control level

^b^SD is the variation of one reference gene across treatments

### Microcystin experiment: Effects on the growth of *Daphnia *magna

Four different treatments were analysed in the microcystin experiment. The green alga *S. obliquus*, a good food-alga, served as calibrator. To account for potential general cyanobacterial effects, *S. elongatus *was included in the experimental design. The aim was to be able to differentiate between the wild type and the mutant of *M. aeruginosa *PCC 7806.

**Figure 4:**
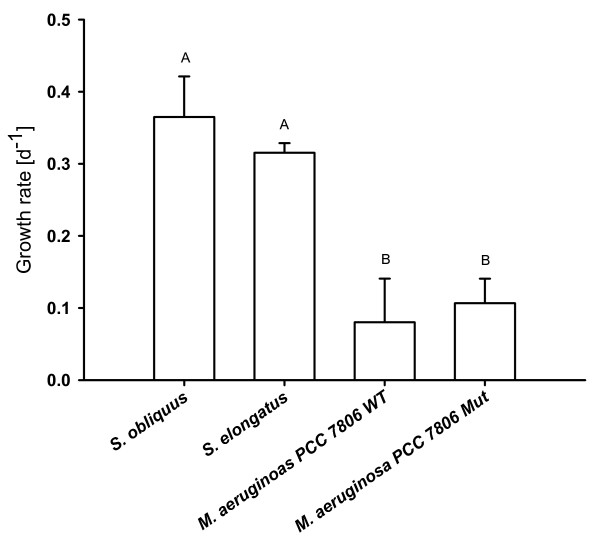
**Growth rates of *D. magna *in the microcystin experiment**. *D. magna *was raised either with pure *S. obliquus*, *S. elongatus *or the wild type (WT) or mutant (Mut) *M. aeruginosa *PCC7806. Depicted is the mean growth rate (n = 3, ± SD) of *D. magna*. Letters indicate a significant difference (p < 0.001) between treatments.

Growth rates of *D. magna *in the *Microcystis *treatments were significantly lower than on *S. obliquus *or *S. elongatus *(Fig. 4; p < 0.001, Tukey's HSD post-hoc, after one-way ANOVA: F_8;0.002045 _= 275.6914, p < 0.001). However, no differences in growth on the wild type or the mutant of *M. aeruginosa *PCC 7806 were observed after four days (Fig. 4). *D. magna *on the wild type strain of *M. aeruginosa *died on day five, whereas all animals kept on the mutant survived.

### Microcystin experiment: Normalisation factors and identification of reference genes

**Figure 5:**
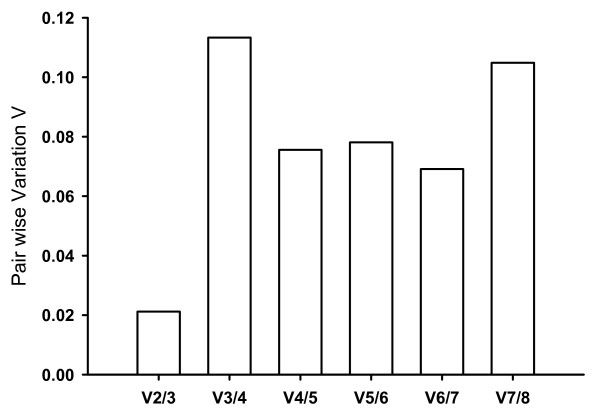
**Pair wise variation of sequential normalisation factors (Vn/n+1) in the microcystin experiment estimated by geNorm**. V2/3 is based on the geometric mean of the normalisation factors of *TBP*, *18S *and *alpha-tubulin*; V3/4 is V2/3 and *SucDH*; V4/5 is V3/4 and *actin*; V5/6 is V4/5 and *GapDH*; V6/7 is V5/6 and *cyclophilin*; V7/8 is V6/7 and *UBC*, V8/9 is V7/8 and *28S*.

The relative expression of nine candidate genes was analysed by geNorm and ranked according to increasing variability (*TBP = 18S < alpha-tubulin < SucDH < actin < GapDH < cyclophilin < UBC < 28S*). GeNorm calculated eight normalisation factors. Pair wise comparison of sequential normalisation factors showed a relatively high level of variability between the three least variable reference genes (V2/3; Fig. 5). The overall effect of using more reference genes was rather small. For this reason, and to simplify experimental handling, only the three least variable genes (*TBP, 18S, alpha-tubulin*) were used as reference genes.

After normalisation, all genes were further analysed. The effects between feeding the wild type or the mutant strain of *M. aeruginosa *PCC 7806 were significantly different (*actin*: Tukey's HSD post-hoc, p < 0.001 after one-way ANOVA: F_8;0.000009 _= 21212.1, p < 0.001; *cyclophilin*: Tukey's HSD post-hoc, p < 0.001 after one-way ANOVA: F_8;0.000060 _= 106222.7, p < 0.001; *GapDH*: Tukey's HSD post-hoc, p < 0.001 after one-way ANOVA: F_8;0.000045 _= 169.04, p < 0.001; *SucDH*: Tukey's HSD post-hoc, p < 0.001 after one-way ANOVA: F_8;0.000010 _= 633236.1, p < 0.001; *UBC*: Tukey's HSD post-hoc, p < 0.001 after one-way ANOVA: F_8;0.000019 _= 87305.5, p < 0.001; *28S*: Tukey's HSD post-hoc, p < 0.001 after one-way ANOVA: F_8;0.000004 _= 671320, p < 0.001;) in every gene. However, the normalised values of the three reference genes showed variation across treatments in the range of 0.3 - 1.0 (Tab. 5), whereas the across-treatment variation was several times higher in three other genes: *GapDH *(4.91); *SucDH *(7.20) and *UBC *(3.79). These three genes of basic metabolism were treated as target genes, and we investigated whether their expression is regulated by the treatments.

**Table 5:** Relative gene expression of the reference genes in *D. magna *after normalisation in the microcystin experiment. *D. magna *was fed either the green alga *S. obliquus *or microcystin-free cyanobacteria (*S. elongatus *or the mutant of *M. aeruginosa *PCC 7806 [Mut]) or the microcystin-producing wild type of *M. aeruginosa *PCC 7806 (WT).

	Relative expression^a^				
Gene	*S. obliquus*	*S. elongatus*	*M. aeruginosa *WT	*M. aeruginosa *Mut	SD^b^
*alpha-tubulin*	1.0000 ± 0.0030	0.3937 ± 0.0021	0.4077 ± 0.0006	0.3028 ± 0.0013	0.3194
*TBP*	1.0000 ± 0.0094	1.0186 ± 0.0056	1.3176 ± 0.0075	3.0758 ± 0.2210	0.9926
*18S*	1.0000 ± 0.0015	2.4935 ± 0.0020	1.8613 ± 0.0073	1.0736 ± 0.0002	0.7079

^a^Values are mean of n = 3 replicates ± SD. Expression levels are displayed relative to the mean control level

^b^SD is the variation of one reference gene across treatments

### Microcystin experiment: Relative expression of the target genes *GapDH*, *SucDH *and *UBC *following normalisation

**Figure 6:**
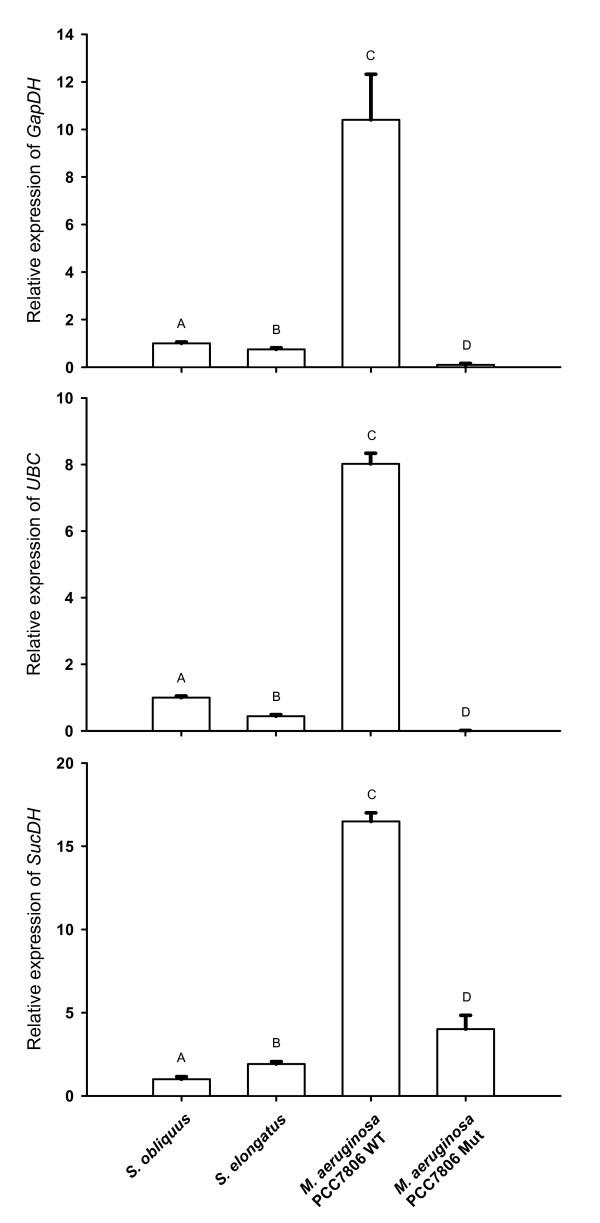
**Relative normalized expression of the target genes *GapDH *(top), *UBC *(middle) and *SucDH *(bottom) in *Daphnia magna *in the microcystin experiment**. *D. magna *was raised either on the green alga *S. obliquus *(= calibrator set at 1), on microcystin-free cyanobacteria (*S. elongatus *or the mutant of *M. aeruginosa *PCC 7806), or on the microcystin-producing *M. aeruginosa *PCC 7806 wild type. Depicted is the mean relative expression (n = 3, ± SD) of *GapDH*, *UBC *and *SucDH*. Letters indicate a significant difference (p < 0.001) between treatments.

The mean relative expression of *GapDH *in the microcystin-free treatments with *S. elongatus*, *S. obliquus *and the mutant of *M. aeruginosa *PCC 7806 ranged between 0.1 and 1 (Fig. 6), whereas the treatment with the microcystin-producing strain of *M. aeruginosa *showed a relative expression of over 10. The pattern of the relative expression of *UBC *was similar. Expression in the treatments with the green alga and the microcystin-free cyanobacteria ranged between 0.01 and 1, whereas the treatment with *M. aeruginosa *wild type showed a relative expression of > 8.0 (Fig. 6). The target gene *SucDH *showed a higher relative expression in the microcystin-free mutant of *M. aeruginosa *(4.0-fold; Fig. 6) than in the microcystin-free treatments with the green alga (1) and *S. elongatus *(1.92); however, the relative expression of *SucDH *in the wild type of *M. aeruginosa *was even 16-folds higher than in the calibrator (green alga).

### Resolving gene identities by homology to the *Daphnia pulex *genome

**Figure 7:**
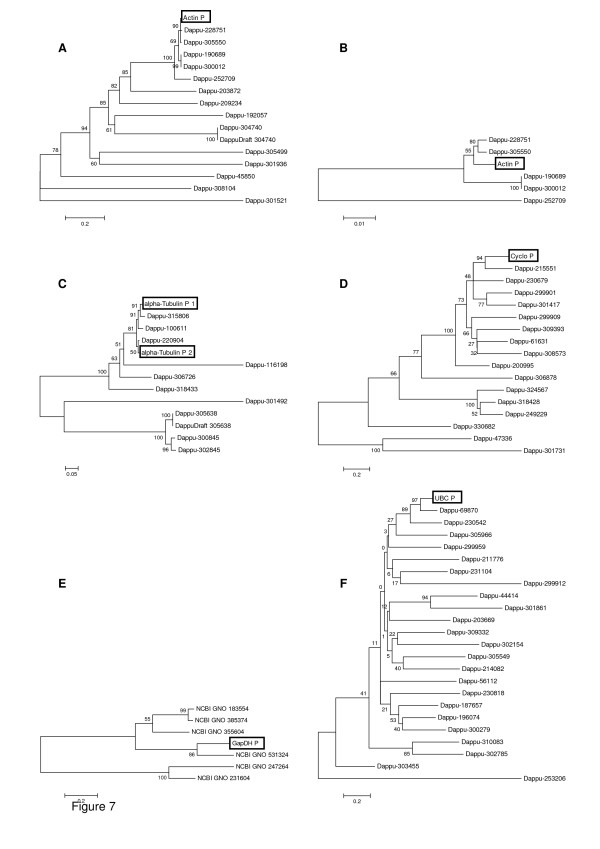
**Phylogenetic trees of the target genes of all experiments**. Neighbor-Joining-Trees with Bootstrap Test of Phylogeny of protein sequences of *D. pulex*. Phylogenetic trees of actin (A) and focus on the cluster of Actin P (B). Phylogenetic trees of alpha-tubulin (C), cyclophiline (D), GapDH (E) and UBC (F). The proteins of the genetic sequences of *D. pulex *equivalent to the utilized *D. magna *sequences are highlighted by boxes. For GapDH genes, no protein ID assignments are given in databases for NCBI Gnomon predicted models 183554 on scaffold 6966:1562-522; 385374 on scaffold 3684:1090-536; 355604 on scaffold 12555:9-671; 531324 on scaffold 2555:31-255; 247264 on scaffold 1546:40515-33280 and 231604 on scaffold 12449:769-1058.

The *D. magna *candidate gene sequences were used for protein database searches for *D. pulex *homologs in the Dappu v1.1 draft genome sequence assembly (September, 2006) and annotation. Phylogenetic trees were subsequently constructed from the aligned amino acids. Fifteen protein sequences for actin could be found. The protein sequence of the orthologous actin in the *D. pulex *sequence (Actin P = Dappu-306442) clustered with five other sequences (Fig. 7A). The highest similarity (55%; p-distance 0.7%) was found with two paralogous sequences (Dappu-228751 and Dappu-305550 (Fig. 7B). P-distances ranged from 0% to 87.7% indicating a very high variability between all of the actin paralogs. Sixteen *D. pulex *proteins with significant sequence similarity to cyclophilin could be found. The cyclophilin protein sequence of the orthologous *D. pulex *sequence (Cyclo P = Dappu-92663) clustered with another sequence (Dappu-215551; p-distance 32%; Fig. 7D). Cyclophilin showed a very high variability with p-distances between 25.2% and 98.1%. For GapDH six significant protein sequence hits could be revealed. The GapDH protein sequence of the orthologous *D. pulex *sequence (GapDH P = Dappu-302823) clustered significantly with another sequence (NCBI GNO 531324; p-distance 34.7%; Fig. 7E). GapDH showed a very high variability with p-distances between 34.7% and 93.9%. Twenty-three significant protein sequence hits for UBC were found. The UBC protein sequence of the orthologous *D. pulex *sequence (UBC P = Dappu-120690) clustered significantly with two other sequences (Fig. 7F). The highest similarity showed Dappu-69870 (97%; p-distance: 19.7%). The variability between paralogs was very high with p-distances between 19.7% and 94.4%. Two different alpha-tubulin loci containing the orthologous EST WFes0007807 from wFleaBase were unearthed. For both loci taken together 11 significant protein sequence hits could be found. The alpha-tubulin protein sequences of the orthologous *D. pulex *sequences (alpha Tubulin P 1 = Dappu-315805 and alpha Tubulin P 2 = Dappu-301837) both clustered significantly with three other sequences (Fig. 7C). Dappu-315806 showed 91% similarity to alpha Tubulin P 1 (p-distance 2.5%), and. Dappu-220904 was similar to alpha Tubulin P 2 (50%; p-distance 3.4%). The variability of alpha-tubulin was very high (p-distances between 2.5% and 77.7%).

## Discussion

The occurrence of cyclic heptapeptides, termed microcystins, is widespread in cyanobacteria and not restricted to the genus *Microcystis*; mass development of microcystin-producing cyanobacteria can constitute a high risk for intoxication of humans and livestock [27]. Numerous studies have been carried out in order to determine the ecological significance of microcystin production in cyanobacteria. The availability of the mcy^- ^mutant of PCC7806, which is genetically identical to the wild type except for its inability to synthesize microcystins [16], made it possible to more precisely analyse the role of microcystins in daphnid poisoning. Life-table experiments with the wild type and the mcy^- ^mutant of PCC7806 with *Daphnia galeata *have shown that the wild type was poisonous to *D. galeata*, whereas the mutant strain had no lethal effects [17,18]. These findings and similar results for *D. magna *[28] suggest that microcystins play a role in the defence of *M. aeruginosa *against zooplankton grazing.

Feeding on the cyanobacterium *M. aeruginosa *led to significantly reduced growth of *D. magna *compared to animals grown on the high quality food alga *S. obliquus *[23] or the non-toxic cyanobacterium *S. elongatus *[22]. Although there was reduced growth in *D. magna *feeding on *M. aeruginosa *compared to the reference cyanobacterium, there was no difference between the wild type and the mcy- mutant treatment. However, a specific microcystin effect became evident on day five, when *D. magna *raised on the wild type strain died, whereas no mortality was observed in *D. magna *raised on the mutant strain.

In an in-vitro system, microcystin-LR has been shown to inhibit protein phosphatases 1 and 2A in crude extracts of *Daphnia *sp [19]. However protein phosphatase 1 and 2A each comprise a family of protein serine/threonine phosphatases with a wide range of different specificities that are mediated by different interactors [29] and regulatory subunits [29,30]. Hence it remains entirely unclear which specific physiological pathways in daphnids are affected by the binding of microcystin to protein phosphatases 1 and 2A.

Here for the first time in-situ effects of dietary microcystins on gene expression of daphnids were investigated. The experiments presented in this paper were designed to identify genes involved in the general metabolism in *D. magna *in which the expression level responds to the presence of microcystins. We therefore compared the effects of the microcystin-producing wild type *M. aeruginosa *PCC7806 and the mcy^- ^mutant of this strain on the relative expression of genes involved in basic metabolism. We found substantial up-regulation of *GapDH *(Dappu-302823) and *UBC *(Dappu-120690) in response to the presence of microcystins in the food of *D. magna*, which demonstrates that certain enzymes of glycolysis and protein catabolism are significantly up-regulated when daphnids ingest microcystins. For the first time a specific gene regulation in response to dietary microcystins has been demonstrated in daphnids. This up-regulation might have enabled *D. magna *to avoid a microcystin-specific depression of growth until day four but could not prevent mortality on day five of the growth experiment.

Upon exposure to the microcystin-producing wild type of *M. aeruginosa *PCC7806, *D. magna *has been shown to develop a tolerance against this toxic strain within an individual's lifespan and to transfer this tolerance to the next generation through maternal effects, a fact that has been interpreted as an inducible defence against microcystin [8]. It remains to be tested which role the observed up-regulation of *GapDH *and *UBC *plays in the inducible tolerance of *D. magna *to microcystins. Furthermore, clones of *D. magna *have been shown to differ in their tolerance to *M. aeruginosa *PCC7806 [31], which suggests a genetic basis for increased toxin tolerance. It remains to be investigated whether the up-regulation of *GapDH *and *UBC *contributes to the tolerance to *M. aeruginosa *PCC7806.

In addition to the microcystins in PCC7806 wild type, both the wild type and mcy^- ^mutant PCC7806 produce other classes of secondary metabolites of unknown biological activity [32,33]. *D. magna *feeding on either of these two strains revealed a substantial up-regulation of *SucDH*, and it remains to be seen which cyanobacterial compounds induce this up-regulation of a key enzyme of the tricarboxylic acid cycle. In order to account for possible general effects of cyanobacteria on expression of the investigated genes, we fed *Synechococcus elongatus *to *D. magna*. This cyanobacterium is easily ingested by daphnids and does not contain toxins or inhibitors [22]. The effects of *S. elongatus *on *GapDH*, *UBC *and *SucDH *were negligible compared to the afore mentioned effects of *M. aeruginosa*, which indicates that the up-regulation of the tested loci of *GapDH*, *UBC *and *SucDH *in *D. magna *is a specific and not a general response to cyanobacterial secondary metabolites. It would be interesting to see, if this holds true for all different paralogs of the affected genes or if the up-regulation is restricted to specific clusters or single paralogs of these highly variable genes (Fig. 7E-F).

Predation is an important stressor in aquatic communities, and many studies using *Daphnia *sp. have contributed to an understanding of the adaptive value of inducible anti-predator defences in the genus *Daphnia*. Achieving a better understanding of the mechanisms and constraints of the evolution of inducible anti-predator defences requires more research on the mechanisms of inducible defences at the molecular level. Only recently has this field been started to be explored. Our work was stimulated by the paper of Pijanowska & Kloc, (2004) [24], who used a clone of *D. magna *which has been shown to be plastic with regard to life-history traits and behaviour [10,11,13,14,34] in response to kairomones from fish and *Chaoborus*. Pijanowska & Kloc (2004) [24] have shown a dramatic decrease of the proteins actin and alpha-tubulin in this clone of *D. magna *when it was exposed to kairomones from planktivorous fish or the larvae of *Chaoborus *water midges. These identical effects of vertebrate and invertebrate kairomones suggested that actin might play a major role in anti-predator responses in *D. magna *in general. Using the same clone of *D. magna*, we here demonstrate that an exposure to chemical cues from both invertebrate and vertebrate predators results in a change in *actin *expression. However, although significant, the 1.75-fold (fish) increase and 0.94-fold (invertebrate) decrease in *actin *expression was rather moderate and did not reflect the dramatic decrease of the protein actin reported by Pijanowska & Kloc [24]. The same holds true for the weak although significant increase in the gene *alpha-tubulin *in the fish (1.71) and the *Chaoborus *treatments (1.07). Since we found two possible alpha-tubulin orthologous protein sequences in *D. pulex*, which were very similar to each other (Fig. 7C), we concluded that the effect on the expression holds true for all paralogs in their cluster. Therefore, the substantial decrease of actin and alpha-tubulin on the protein level reported by Pijanowska & Kloc [24] could be a posttranslational process, e.g. miRNA-mediated regulation or increased degradation, as has been suggested by the authors [24]. We conclude that these loci of *actin *and *alpha-tubulin *are no strong target genes for anti-predator defences. However, construction of phylogenetic trees reveals very high variability between the different paralogs of actin and alpha-tubulin (Fig. 7A-C). It remains to be tested if the decrease of actin and alpha-tubulin on the protein level reported by Pijanowska & Kloc [24] is caused by another paralogous sequence sharing the same gene name.

Following normalisation to NF, it turned out that the expression of *28S*, *UBC*, *18S *and *cyclophilin *was affected by the type of kairomone. Genes involved in protein biosynthesis (*18S, 28S*) and protein catabolism (*UBC*) were up-regulated by kairomone. These effects were considerably stronger for fish kairomone. The expression of *cyclophylin *(Dappu-92663), a gene involved in protein folding, was up-regulated in the presence of kairomones from vertebrate and down-regulated by kairomones from invertebrate predators. The finding that the two kairomones differ in their effect on *cyclophylin *in *D. magna *is in accord with the observation that the life-history response of this clone of *D. magna *differs between kairomones released from fish or *Chaoborus *[24]. *Cyclophilin*, could serve as a potential target gene for further analysis of kairomone effects on daphnids. It remains to be seen how *cyclophilin *is involved in mediating kairomone effects on life history of daphnids and if this is specific to the orthologous sequence and to related paralogous sequences of *cyclophilin*.

Our study is the first detailed study that investigates effects of kairomones from vertebrate and invertebrate predators and of microcystin on gene expression of genes involved in different basic metabolic processes in *D. magna*. Kairomones from both vertebrate and invertebrate predators led to the well-established adaptive shifts in SFR in *D. magna *giving evidence for biologically active incubation water from either predator. Similarly, evidence for specific effects of microcystin comes from the higher mortality of *D. magna *on the wild type strain than on the mutant of *M. aeruginosa *PCC 7806. Calculating a combination normalisation factor based on the geometric mean of three genes for the kairomone experiment and for the growth experiment, stressor-specific regulation of some of the genes involved in basic metabolism is demonstrated.

All target genes in *Daphnia *show a surprisingly high variability between paralogs. If such a high variability holds true for other genes in *D. magna*, this could hint at a highly plastic genome, which might be adaptive for an animal that living in a very complex aquatic environment and therefore has to maintain a high potential for adaptations.

## Conclusion

Three (*GapDH, TBP, SucDH*) of the seven genes investigated (*GapDH, TBP SucDH, 28S, UBC, 18S, cyclophilin*) were found to be stable across the kairomone treatments and were used as reference genes for normalization. Although significant, no substantial kairomone-dependent regulation of *actin *and *alpha-tubulin *was found, indicating that the dramatic decrease of actin and alpha-tubulin at the protein level in response to kairomones (reported earlier) was not due to a regulation of the transcription of the *actin *and *alpha-tubulin *loci investigated here. Therefore these gene loci cannot serve as target genes in the analysis of kairomone effects on *D. magna*. If this holds true for the other paralogs sharing the same gene name remains to be tested. However, the expression of other genes involved in protein biosynthesis, protein catabolism and protein folding, especially the regulation of *cyclophilin *by kairomones, indicated major effects on protein folding. These genes have the potential to serve as target genes in further analysis of kairomone effects on the life history of daphnids.

Three genes (i.e. *18S, TBP, alpha-tubulin*) proved to be stable across microcystin-free and microcystin-containing cyanobacterial food treatments and were used for normalization. Two of the candidate genes (*UBC *and *GapDH*) were shown to have toxin-specific regulation and were clearly up-regulated when microcystins were present in the food. This indicates that microcystins strongly affect protein catabolism and glycolysis in *D. magna *when the animals ingest microcystins via the natural route of exposure, i.e. uptake of microcystin-containing food items; it remains to be seen which role the observed up-regulation of *GapDH *and *UBC *plays in the inducible tolerance of *D. magna *to microcystins.

The construction of phylogenetic trees is an essential step in target gene analysis in *Daphnia *in order to account for the high variability between different paralogs. Phylogenetic trees of the different paralogs are indispensable for clustering the utilized loci with similar ones and to delineate them from others. Related paralogs might have a similar relevance within the genome. This approach is especially important in an organism like *Daphnia sp*. with p-distances showing a very high variability between different paralogs.

## Methods

### Test species and cultures

A clone of *Daphnia magna *originating from Lake Binnensee, Germany, inhabited by fishes and various invertebrates, was cultured at 20°C in membrane-filtered tap water (conductivity: 740 μS/l; pH 7.2; major ions: Ca^2+ ^(110 mg/l) and HCO^3- ^(270 mg/l)).

Fifteen animals per litre were kept under non-limiting food concentrations (2 mg C_part_/l) with *Scenedesmus obliquus *(SAG-276-3a) (Stammsammlung für Algen, Göttingen, Germany) as food alga. Only third clutch neonates which had been born within 12 h were used for the experiments.

The green alga *Scenedesmus obliquus*, the cyanobacteria *Synechococcus elongatus *(SAG 89.79) and *Microcystis aeruginosa *(UTEX LB 2063 and PCC 7806), and a genetically engineered microcystin synthetase knock out mutant of *Microcystis aeruginosa *(PCC 7806 mcy^- ^[16]) were cultivated semi-continuously in cyanophyceen medium [35] at 20°C, with half of the medium exchanged weekly. The medium consisted of 0.6 mM CaCl_2 _× 2 H_2_0, 0.8 mM NaNO_3_, 0.4 mM K_2_HPO_4 _× 3 H_2_O, 0.4 mM MgSO_4 _× 7 H_2_O, 0.01 mM NaFeEDTA, 0.8 mM KCl, 100 μM H_3_BO_3 _and 20 μM Na_2_MoO_4 _× 2 H_2_O and had a pH of 8.5. Cyanobacteria were cultivated with constant light at 95 μE, *S. obliquus *at 130 μE. Carbon concentrations of the autotrophic food suspensions were estimated from photometric light extinction (800 nm) and from carbon-extinction equations previously determined.

### Experimental design

#### Kairomone experiment

Fish-conditioned water was prepared by exposing four sunbleaks (*Leucaspius delineatus*) in 4 l of tap water at 20°C for 24 h without feeding during the whole experiment. The fishes were stopped being fed for 24 h prior to the experiment; the water thus did not contain any faeces.

The *Chaoborus *incubation water was prepared by exposing 60 fourth-instar larvae of *Chaoborus flavicans *(which had previously been allowed to feed on zooplankton for 4 h each day of the experiment) in 1 l of tap water at 15°C for 20 h.

Predator-conditioned water was filtered (GFF), and in case of fish-conditioned water diluted 1:4, prior to introducing *D. magna*. From a cohort of *D. magna *neonates that had been born within 12 h from synchronized mothers, five animals each were exposed in 250 ml glass containers to predator-conditioned or control water from birth until maturity. All treatments were run in triplicate. *D. magna *were fed the green alga *S. obliquus *(4 mg Cpart/l); the media were changed daily. At the day when the 1st clutch was visible size at first reproduction (SFR) was determined as the size of the egg-bearing *D. magna*. For each replicate a mean SFR was calculated, and these mean values were used to calculate the respective mean value and the variance for the treatment.

#### Microcystin experiment

From a cohort of new born *D. magna*, 8-10 animals each were transferred to 1 l of tap water with a food concentration of 2 mg C_part_/l. The animals were either fed the green alga *S. obliquus *as a control for high quality food or one of the three cyanobacteria. Each day the medium and the food were exchanged. The experiment took place under low light conditions at 20°C and lasted for four days for the real-time PCR analysis. All food treatments were run in triplicate, and somatic growth rates of *D. magna *were determined from dry weight of animals collected at the start and at day four of the experiment. according to [23].

#### RNA extraction and reverse transcription

At the end of the experiments the animals' RNA was extracted using the RNeasy Mini Kit (Qiagen). In order to remove any traces of genomic DNA, the RNA was treated with Desoxyribonuclease I (Fermentas) following the manufacturer's instructions. The integrity of the RNA was verified with 1.5% agarose gel electrophoreses. RNA concentrations were determined with a Qubit Fluorometer (Invitrogen). 1 μg of RNA was reverse transcribed using the High Capacity cDNA Reverse Transcription Kit (Applied Biosystems). The cDNA was diluted 50-fold resulting in total RNA concentrations of 1 ng/μl. The cDNA was stored at -20°C.

#### Quantitative real-time PCR (QPCR)

Nine different housekeeping genes recently introduced for QPCR in *D. magna *by Heckmann et al. (2001) [26] were used in QPCR analysis: *actin, alpha-tubulin, cyclophilin, glyceraldehyde-3-phosphate dehydrogenase (GapDH), succinate dehydrogenase (SucDH), TATA-box binding protein (TBP), ubiquitin conjugating enzyme (UBC), 18S ribosomal RNA (18S), 28S ribosomal RNA (28S)*.

QPCR was conducted on the 7300 real time PCR system (Applied Biosystems). Each reaction contained 5 μl of cDNA template, 10 μl Power SYBR^® ^Green PCR Master Mix (Applied Biosystems) and 2.5 μM of each primer in a final volume of 20 μl. Each reaction was conducted in triplicate. Cycling parameters were 95°C for 10 min to activate the DNA polymerase followed by 40 cycles of 95°C for 15 s and 60°C for 1 min. After the actual analysis, dissociation curves were performed to verify that no primer-dimers had been amplified. Outliers and samples diverging from the dissociation curve were omitted.

#### Data analysis and statistics

The raw data were analysed after QPCR. Because of the differing amplification efficiencies of the primer pairs [26], the relative expressions were calculated as quantities using the formula W_R _= (E+1)(C_Tmin_-C_Tx_) (a modification of the 2^-ΔΔC^_T _Method [36]), in which W_R _is a quantity for the relative expression of one sample, E is the amplification efficiency of its assay, C_Tmin _is the lowest threshold cycle of all samples of this assay, and C_Tx _is the threshold cycle of the analysed sample. The quantities could then be imported into geNorm version 3.4 [25], an Excel (Microsoft) based tool which calculates the minimum required number and best-suited combination from a given set of reference genes and from that generates a "normalisation factor" to be used for stable normalisation of QPCR measurements.

After determination of a normalisation factor from the set of reference genes, the raw data of the QPCR runs were imported into qBase version 1.3.5 [37], an Excel (Microsoft) based tool which calculates relative gene expression normalised using the normalisation factor generated with geNorm. *D. magna *fed entirely with *S. obliquus *served as calibrator which was always set as 1.

The growth rates and the body length were (×2)^-1 ^transformed when needed to ensure homogeneity of variances and analysed with ANOVA and Tukey's honestly significant difference (HSD) for post hoc comparisons to assess differences in relative expression.

The data generated with qBase were log-transformed when needed to ensure homogeneity of variances and analysed with ANOVA and Tukey's honestly significant difference (HSD) for post hoc comparisons to assess differences in relative expression.

The statistics were performed with Statistica 6.0.

#### Database search and construction of phylogenetic trees

To compare the *Daphnia magna *sequences with the *Daphnia pulex *database http://wfleabase.org and to discover homologs in the genome for the genes of interest, the *D. magna *sequences of the target genes *actin, alpha-tubulin, cyclophilin, GapDH *and *UBC *[26] were used as queries for sequence similarity searches using BLASTn against the Dappu v1.1 draft genome assembly in wFleabase. The best alignments with the highest score was taken as the ortholog for the sequence of interest in *D. pulex*. Its protein sequence was subsequently used to query for additional homologs using BLASTp (blastp; e-value cut off limit = 0.01) against the the v1.1 gene builds (July, 2007) archived in wFleaBase. All protein sequences with a significant hit were taken and aligned (BioEdit v.7.0.5.3 [38]) with the orthologous protein sequence. Using the program MEGA 4 [39] Neighbor-Joining trees with Bootstrap Test of Phylogeny were constructed and p-distances were calculated. Hypothetically, the *D. pulex *trees and the *D. magna *trees are approximately identical.

## Abbreviations

C_part_: particulate organic carbon; GapDH: glyceraldehyde-3-phosphate dehydrogenase; SucDH: succinate dehydrogenase; TBP: TATA-box binding protein; UBC: ubiquitin conjugating enzyme; 18S: 18S ribosomal RNA; 28S: 28S ribosomal RNA.

## Authors' contributions

EVE and AS designed and coordinated the study. AS performed all practical aspects under the supervision of EVE and CC. The work was supported by a grant to EVE (DFG EL 179/6-1). All authors read, contributed and approved the final manuscript.
